# Detection and Treatment with Emetine Bismuth Iodide of Amebic Dysentery Carriers among Cases of Irritable Heart

**Published:** 1918-05

**Authors:** 


**Detection and Treatment with Emetine Bismuth Iodide of Amebic Dysentery Carriers among Cases of Irritable Heart.** By Margaret W. JEPPS and J. C. MEAKINS, Major C. A. M. C. (Report to the Medical Research Committee), *Brit. Med. Jour.*, Nov. 17, 1917.

65 cases with irritable heart were examined, of which 25 had a definite history of dysentery. The authors conclude from their investigations that :

1. Additional infections with intestinal protozoa may be detected at the sixth and later examinations.

2. Infections of E. histolytica in which no cysts measuring over 10 μ in diameter are found in the stools are far from uncommon.

3. The use of emetine bismuth iodide has cured a large proportion (95 per cent.) of our amebic dysentery carriers.

4. The best methods of administration of this drug is in the form of the loose powder in cachets in daily doses of 3 grains. At least 36 grains should be given in all.

5. Symptoms of irritable heart in amebic dysentery carriers are reduced in a large number of those patients who have been cured of the amebic infection.

6. Infection with intestinal protozoa has no marked effect on the differential leucocyte count.

